# Comparability of Gastrointestinal Microbiome and Bile Acid Profiles in Patients With First or Multiply Recurrent *Clostridioides difficile* Infection

**DOI:** 10.1093/infdis/jiaf408

**Published:** 2025-08-02

**Authors:** Jessica A Bryant, Timothy J Straub, Darrell S Pardi, Kevin D Litcofsky, Colleen R Kelly, Meghan E Chafee, Stuart H Cohen, Sahil Khanna, Charles S Berenson, Jennifer Wortman, Matthew Sims, Christopher B Ford, Mary-Jane Lombardo, Barbara H McGovern, Lisa von Moltke, Colleen S Kraft, Matthew R Henn, Brooke R Hasson

**Affiliations:** Research and Development, Seres Therapeutics, Cambridge, Massachusetts, USA; Research and Development, Seres Therapeutics, Cambridge, Massachusetts, USA; Division of Gastroenterology and Hepatology, Mayo Clinic, Rochester, Minnesota, USA; Research and Development, Seres Therapeutics, Cambridge, Massachusetts, USA; Division of Gastroenterology, Brigham and Women's Hospital, Boston, Massachusetts, USA; Research and Development, Seres Therapeutics, Cambridge, Massachusetts, USA; University of California Davis Health, Division of Infectious Diseases, Sacramento, California, USA; Division of Gastroenterology and Hepatology, Mayo Clinic, Rochester, Minnesota, USA; University at Buffalo, Department of Medicine, Jacobs School of Medicine & Biomedical Sciences, VA Western New York Healthcare System, Buffalo, New York, USA; Research and Development, Seres Therapeutics, Cambridge, Massachusetts, USA; Section of Infectious Diseases and International Medicine, Department of Internal Medicine, Beaumont Royal Oak, Royal Oak, Michigan, USA; Departments of Internal Medicine and Foundational Medical Studies, Oakland University William Beaumont School of Medicine, Rochester, Michigan, USA; Research and Development, Seres Therapeutics, Cambridge, Massachusetts, USA; Research and Development, Seres Therapeutics, Cambridge, Massachusetts, USA; Research and Development, Seres Therapeutics, Cambridge, Massachusetts, USA; Research and Development, Seres Therapeutics, Cambridge, Massachusetts, USA; Department of Pathology and Laboratory Medicine, Division of Infectious Diseases, Emory University, Atlanta, Georgia, USA; Research and Development, Seres Therapeutics, Cambridge, Massachusetts, USA; Research and Development, Seres Therapeutics, Cambridge, Massachusetts, USA

**Keywords:** microbiome, microbiome therapeutic, *Clostridioides difficile* infection, SER-109, VOS

## Abstract

**Background:**

*Clostridioides difficile* infection (CDI) treatment guidelines suggest varied approaches for patients with first (frCDI) or multiply recurrent CDI (mrCDI). Low microbial diversity, elevated primary bile acids (BA), and low secondary BA concentrations favor germination of *C*. *difficile* spores into toxin-producing bacteria and are believed to increase rCDI risk. Greater understanding of the gastrointestinal (GI) microbiome in rCDI may inform management of the disease. We describe a post hoc comparison of GI microbiome and bile acid profiles between patients with frCDI and mrCDI in a Phase 3 open-label trial, ECOSPOR IV, of fecal microbiota spores, live-brpk (VOWST®; VOS, formerly SER-109), an orally-administered live microbiome therapeutic.

**Methods:**

Patients received VOS following symptom resolution after standard-of-care antibiotics. Pretreatment baseline (within 3 days following antibiotic completion) and Week 1 post-dosing stool samples were collected for whole metagenomic sequencing and metabolomics. Diversity was calculated from MetaPhlAn2 species profiles. Concentrations of primary and secondary BAs were measured via targeted LC-MS/MS.

**Results:**

rCDI rates through Week 8 were similarly low in both frCDI and mrCDI patients (6.5% versus 9.7%, respectively). Baseline microbial diversity was similarly low between frCDI and mrCDI subgroups (*P* > .05). Diversity and secondary BA concentrations increased in both subgroups, whereas primary BA concentrations declined following VOS dosing, leading to few differences between subgroups at Week 1.

**Conclusions:**

These data suggest commonalities in microbiome disruption in patients with frCDI and mrCDI that contribute to recurrence and suggest that antibiotics followed by a live microbiome therapy may be an optimal treatment strategy for rCDI, regardless of number of prior CDI recurrences.

## Teaser

This study finds commonalities in microbiome disruption of patients with first and multiply recurrent CDI suggesting that antibiotics followed by a live microbiome therapy may be an optimal treatment strategy for rCDI, regardless of number of prior CDI recurrences.

Most patients with primary *Clostridioides difficile* infection (CDI) attain a sustained clinical response after treatment with antibiotics, but 20–25% rapidly develop recurrence within 8 weeks of antibiotic discontinuation. Patients with a history of recurrence have >40% risk for further episodes [1, 2]. Greater understanding of the pathogenesis of recurrent CDI is important for prevention and treatment of the disease. Many factors increase risk for recurrence including older age and the presence of comorbidities, but a common underlying determinant is gastrointestinal (GI) microbiome disruption. The loss of microbial diversity and altered microbe-associated metabolites within a disrupted microbiome support conditions favorable to the germination of *C. difficile* spores into toxin-producing bacteria that cause colitis with debilitating diarrhea. Current antibiotic treatments for rCDI do not address the underlying GI disruption and can promote further disruption, supporting a cycle of recurrence, rather than a lasting therapeutic effect [3, 4]. Fecal microbiota spores, live-brpk (VOWST®; VOS; formerly known as SER-109) is an FDA-approved orally administered consortium of purified Firmicutes (renamed Bacillota) bacterial spores indicated to prevent the recurrence of CDI in adults ≥18 years of age following antibacterial treatment for recurrent CDI. In the randomized, double-blind Phase 3 ECOSPOR III trial, CDI recurrence was significantly reduced at 8 weeks in patients who received VOS compared with placebo following antibacterial treatment (12% versus 40%, respectively, relative risk, 0.32 [95% CI, 0.18–0.58; *P* < .001]) [1]. In a larger, open-label study (ECOSPOR IV) of patients with first or multiply recurrent CDI treated with VOS, a similarly low overall rate of recurrence (9%) was observed [5]. In both trials, VOS was well-tolerated.

Several microbe-associated metabolic pathways such as bile acid and short chain fatty acid metabolism are likely important in supporting the two-phase life cycle of *C. difficile* [6, 7]. In vitro and in vivo evidence is substantive regarding the role of bile acids. Primary bile acids facilitate *C. difficile* spore germination into replicating vegetative bacteria while secondary bile acids are important in limiting vegetative bacterial replication and growth [8–10]. Preplanned exploratory microbiome analyses of patients in ECOSPOR III showed that primary bile acids are elevated at baseline (within 3 days following antibiotic completion), while secondary bile acids are depleted [1, 11]. However, compared with placebo, VOS treatment led to rapid and significant increases in secondary bile acid concentrations and reduced primary bile acid concentrations, a rebalancing which is thought to be critical to the durable clinical response [1, 11]. These observations support the distinct roles of primary and secondary bile acids in modulating the *C. difficile* life cycle and the positive impact of Firmicutes spores in restoring *C. difficile* colonization resistance.

CDI management guidelines suggest a variety of treatment algorithms based on whether the patient has a first, second, or third (or further) recurrence [12–14]. Options include (1) selecting an alternative antibiotic that was not used for the primary episode, (2) the addition of bezlotoxumab (monoclonal antibody against *C. difficile* toxin B) to standard-of-care antibiotics, (3) pulse-taper antibiotic regimens, and (4) unapproved fecal microbiota transplantation (FMT) for those with a history of ≥2 recurrences. However, sustained clinical response rates remain low in the setting of recurrence and treatment with antibiotics alone can further exacerbate the disrupted microbiome, increasing risk for recurrence. Studies of FMT highlight that microbiome restoration is key to a sustained clinical response. However, due to limited processing, FMT can serve as a transmission vehicle for undetected infectious agents (ie, viruses, fungi, and Gram-negative bacteria) leading to hospitalization and death [15]. Recently published clinical practice guidelines from the American Gastroenterological Association (AGA) also suggests use of FDA-approved VOWST and REBYOTA® (fecal microbiota, live-jslm; formerly RBX2660) be considered in immunocompetent adults after the second recurrence, including in select patients after first CDI occurrence if they have a high risk of recurrence or morbid CDI recurrence (acknowledging the opportunity to prevent a first recurrence in these at risk patients) [14].

Enhanced understanding of whether the pathogenesis of first recurrence (frCDI) is similar to that of multiply recurrent infection (mrCDI) may help inform whether other patients may also benefit from earlier use of microbiota-based therapeutics, such as VOS. In one small study, patients with recurrent CDI had markedly lower microbial diversity than those with primary infection [16]. Another study reported significant differences in bile acid profiles in patients who had experienced 3 or more episodes of CDI versus those with primary CDI [17, 18]. However, there are scarce data as to whether the profile of the GI microbiome and metabolome varies between patients with first or multiply recurrent CDI.

We previously reported in ECOSPOR IV, a single-arm, open-label Phase 3 trial with VOS, which included patients with a history of first and multiply recurrent CDI, CDI recurrence rates at Week 8 were similarly low in both frCDI and mrCDI patients (6.5% [95% CI, 2.1%–14.5%] and 9.7% [95% CI, 5.8%–14.9%], respectively) [5]. In this post hoc analysis using whole metagenomics sequencing (WMS) and targeted metabolomics, we compared baseline and post-dosing microbiome and bile acid profiles, between patients with frCDI and mrCDI to improve understanding of rCDI pathology [5].

## METHODS

ECOSPOR IV was an open-label single arm study conducted at 72 US and Canadian sites from October 2017 to April 2022 (NCT03183141; registration date: 08 June 2017). The trial was performed in accordance with Good Clinical Practice (GCP) and the protocols and amendments were reviewed and approved by local or central investigational review boards. Written informed consent was obtained from participants at screening.

### Study Participants and Procedures

A total of 263 patients with recurrent CDI were enrolled in 2 cohorts: Cohort 1 included rollover patients from the Phase 3 randomized, placebo-controlled trial, ECOSPOR III, who experienced on-study recurrence diagnosed by toxin EIA (N = 29); and Cohort 2 included de novo patients with ≥1 CDI recurrence (diagnosed by PCR or toxin EIA), inclusive of the current episode (*N* = 234). Open-label investigational product (VOS) was administered orally as a dose of 4 capsules per day over 3 consecutive days following symptom resolution after standard-of-care antibiotics. Antibiotic selection (vancomycin or fidaxomicin) for the acute CDI episode was at the discretion of investigators. Patients were instructed to take a laxative 1 day prior to VOS treatment initiation to reduce residual antibiotic in the GI tract. Patients were monitored for up to 24 weeks for CDI recurrence. Additional details of the trial design are published elsewhere [5].

Microbiome analyses were conducted using only Cohort 2 due to deviations from recommended timing between antibiotic and VOS treatment in Cohort 1. Stool samples were collected pretreatment (baseline within 3 days following completion of antibiotic treatment for the qualifying CDI episode), and patients were asked to provide an optional additional stool sample at Week 1 post-treatment. Samples were collected in tubs and shipped on frozen gel packs to a central laboratory where they were homogenized, aliquoted, and then immediately frozen at −80°C. Aliquots of 12% weight/weight stool suspensions in 95% ethanol were used for WMS and neat aliquots were used for bile acid measurements.

Microbiome species profiles were generated from preprocessed, sequence depth normalized WMS data as previously described using MetaPhlAn2 with a proprietary database of species markers for consistency with previous publications [1, 19–21]. A quantitative assessment of key primary (cholic (CA) and chenodeoxycholic acid (CDCA)) and secondary (deoxycholic (DCA), lithocholic (LCA), and ursodeoxycholic acid (UDCA)) bile acids in stool were measured with on homogenized, lyophilized stool samples using chromatography/mass spectrometry (LC-MS/MS) analysis with an Agilent 1290/Sciex 5500 QTRAP system equipped with an Agilent SB-C18 reversed phase column (Metabolon, Durham, NC, USA). PERMANOVA tests were run to compare microbiome composition across study groups using the adonis2 function in the vegan package [22]. Alpha diversity and bile acid concentrations comparisons were carried out with two-sided Wilcoxon rank-sum tests (MWU), treating antibiotic as a conditional variable using the wilcox_test function in the coin package (R version 3.6.0, [23, 24]). See supplementary methods for additional details.

Microbiome data for both treatment arms (VOS and placebo) in the randomized, placebo-controlled ECOSPOR III trial (all mrCDI patients; Feuerstadt et al. [1]) are presented graphically for visual comparison.

## RESULTS

A total of 186 patients had a history of ≥2 prior episodes of CDI (mrCDI), and 77 patients were enrolled with their first recurrence of CDI (frCDI) [1, 4]. Detailed demographics have been previously published [1, 5]. At least one stool sample was available for microbiome analyses from 69 patients with frCDI and 142 patients with mrCDI from Cohort 2; hereafter, the “microbiome population”. Age was similar in both frCDI and mrCDI patients (mean age: 61.5 years and 64.0 years, respectively). Use of vancomycin was highly prevalent in the microbiome population and was administered in 68.1% of patients with frCDI and 73.9% of patients with mrCDI [1, 5].

**Figure 1.**
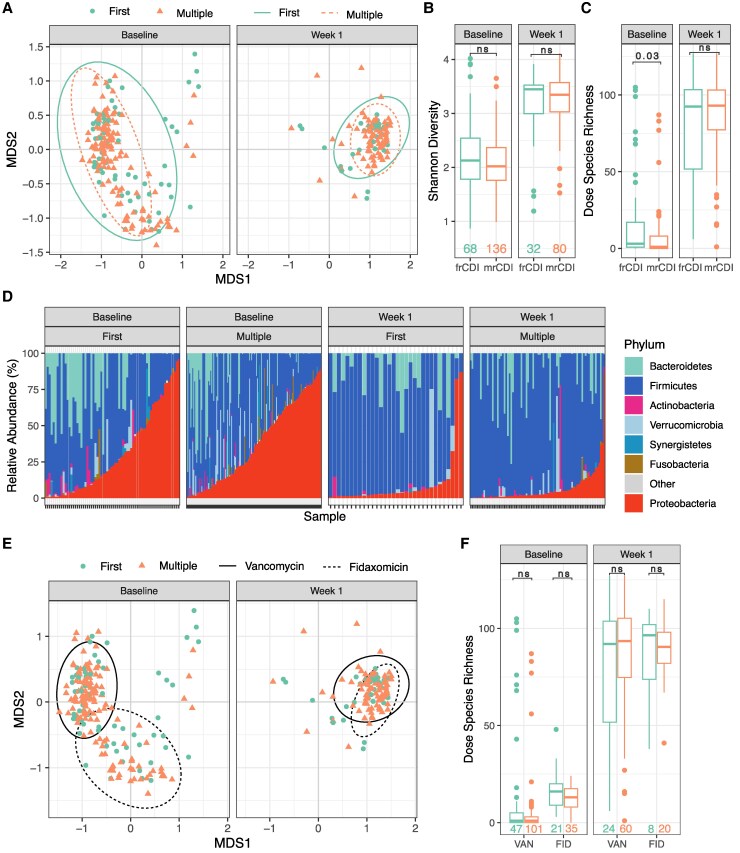
Alpha and beta diversity of first (frCDI) versus multiply (mrCDI) recurrent CDI patient microbiomes at baseline and Week 1. *A*, NMDS plots visualizing Bray–Curtis dissimilarities between samples (stress = 0.15, k = 3, see Supplementary Figure 1 for additional axis). Circular points indicate frCDI and triangles indicate mrCDI samples. Ovals indicate 95% confidence ellipses encircling frCDI and mrCDI samples with solid and dashed lines, respectively. Box plots displaying (*B*) Shannon diversity and (*C*) dose species richness show the median value (central horizontal line) with interquartile range (box). Vertical bars indicate the most extreme non-outlier values (within 1.5 times the interquartile range), and points indicate outlier values (outside 1.5 times the interquartile range). *D*, Bar chart displaying the relative abundances of major bacterial phyla across samples, across timepoints and recurrence status. Samples are ordered along the x-axis by the relative abundance of Proteobacteria. *E*, Copy of NMDS plot in (A), with 95% confidence ellipses circling patients who were treated with either vancomycin (solid line) or fidaxomicin (dashed line) for SoC treatment in their qualifying episode of rCDI prior to VOS dosing. *F*, Box plot displaying dose species richness as done in Figure 1*C*, but separating patients who received SoC vancomycin and fidaxomicin. Sample numbers for all figures are displayed in text on the bottom of Figure 1*B* and 1*F*. ns, not significant, *P* > .05.

An initial examination of the overall microbiome dataset structure (Figure 1*A*) was conducted to support sound statistical analyses of downstream frCDI versus mrCDI comparisons. NMDS plots in combination with modeling variability in microbiome communities by time point and select baseline characteristics, age, sex, antibiotic used to treat the qualifying episode of rCDI completed just prior to VOS treatment, diagnostic test used for the qualifying CDI episode, and frCDI versus mrCDI [5], revealed that the largest proportion of variance in community composition was explained by time point followed by antibiotic (Figure 1*A* and 1*E*, PERMANOVA, *P* < .001, Supplementary Table 1). Variance explained by frCDI versus mrCDI, was an order of magnitude lower and not statistically significant (*P* > .05).

### Baseline Microbiome and Bile Acid Profiles in frCDI vs mrCDI Patients

Analysis of microbiome community composition within baseline samples before treatment with VOS did not reveal significant differences between frCDI and mrCDI patients (Figure 1*A* and 1*D*, PERMANOVA, *P* > .05, Supplementary Figure 1, Supplementary Table 2]). Similarly, no significant differences were observed at baseline using species richness or Shannon diversity metrics (MWU *P* > .05, Figure 1*B*, Supplementary Figure 2, Supplementary Table 3). Dose species richness was significantly different between frCDI and mrCDI patients but differences were numerically small (median number of dose species: 3 versus 1 in frCDI and mrCDI, respectively) and the interquartile ranges of the two groups largely overlapped (MWU, *P* = .03, Figure 1*C* and 1*F*, Supplementary Figure 2, Supplementary Table 3).

**Figure 2.**
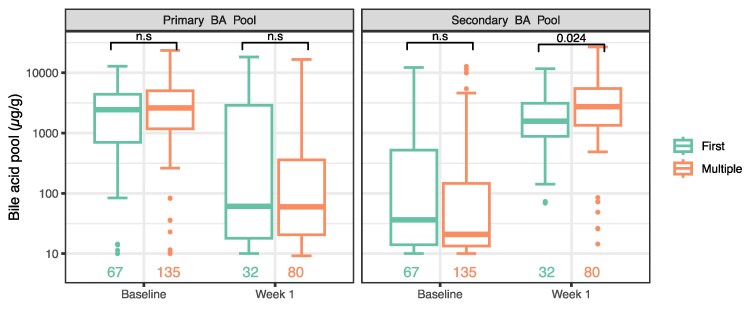
Primary and secondary bile acid concentrations in first (frCDI, first box in green) versus multiply (mrCDI, second box in orange) recurrent CDI patients at baseline and Week 1. *A*, Pooled primary bile acid (CA and CDCA) concentrations (in µg/g) for patients treated with VOS. *B*, Pooled secondary bile acid (LCA, DCA, and UDCA) concentrations (in µg/g) for patients treated with VOS. Sample numbers are displayed in text on the bottom of each figure. *P* values indicate MWU tests comparing frCDI versus mrCDI. ns, not significant, *P* > .05.

An evaluation of whether individual species or genera were more prevalent or had higher relative abundances in frCDI versus mrCDI patients at baseline revealed that no species demonstrated significant associations (MaAslin3, False Discovery Rate > 0.1). Similarly, no significant differences were observed in individual or pooled primary and secondary bile acid concentrations between frCDI and mrCDI patients at baseline (MWU, *P* > .05, Figure 2, Supplementary Figure 3, Supplementary Table 4).

### Change From Baseline to Week 1 in frCDI vs mrCDI Patients

Microbiome composition significantly shifted between baseline and Week 1 time points, as visualized by the nearly complete separation of baseline and Week 1 samples across the x axis of the NMDS plots (Figure 1*A* and 1*E*, PERMANOVA, *P* < .001, Supplementary Table 5). The magnitude of the changes in community composition, quantified with Bray-Curtis distances between paired patient baseline and Week 1 samples, were similar between frCDI and mrCDI patients (MWU, *P* > .05, Supplementary Figure 4, Supplementary Table 2).

The large change in microbiome community compositions from baseline to Week 1 corresponded with a large increase in all alpha diversity metrics including dose species richness (MWU, *P* < .001, Figure 1*B*, 1*C*, and 1*F*, Supplementary Figure 2, Supplementary Table 6). Consistent with this finding, we observed strong VOS engraftment in both frCDI and mrCDI patients, as defined by the number of newly appearing dose species at Week 1 (Supplementary Figure 2). The number of newly appearing dose species observed in frCDI versus mrCDI at Week 1 was not significantly different (*P* > .05, Supplementary Table 2).

The large taxonomic changes corresponded with significant changes in bile acids. Individual and pooled primary bile acid concentrations decreased from baseline to Week 1 in both frCDI and mrCDI patients (Figure 2, Supplementary Figure 3, Supplementary Table 7). Conversely, individual and pooled secondary bile acid concentrations were low at baseline and increased post-VOS dosing in both frCDI and mrCDI patients (Figure 2, Supplementary Data Figure 3, Supplementary Table 7). All changes in bile acid concentrations between baseline and Week 1 were significant (MWU, *P* < .05, Supplementary Table 7).

### Post-dosing Microbiome and Bile Acid Profiles in First vs Multiply Recurrent Patients

Analysis of community composition within Week 1 samples, following VOS treatment, did not reveal differences between frCDI and mrCDI patients (Figure 1*A*, PERMANOVA, *P* > .05, Supplementary Figure 1, Supplementary Table 1). Furthermore, there were no observed differences between frCDI and mrCDI patients at Week 1 when considering any of the three alpha diversity metrics (Figure 1*B*, 1*C*, and 1*E*, Supplementary Data  Figure 2, Supplementary Table 3, MWU > 0.05), nor did regression analyses identify any significant associations between individual species prevalence or relative abundances and frCDI versus mrCDI groupings (MaAslin3, False Discovery Rate > 0.1).

Individual and pooled primary bile acid concentrations were comparable between frCDI and mrCDI patients at Week 1 (MWU *P* > .05, Figure 2, Supplementary Figure 3, Supplementary Table 4). The median values of pooled secondary bile acids and individual secondary bile acids DCA and LCA, were roughly 40% lower in frCDI patients compared with mrCDI patients (MWU *P* < .05, Figure 2, Supplementary Figure 3, Supplementary Table 4), although there were substantial overlaps in the interquartile ranges of secondary bile acid concentrations between the two groups. Note the differences in frCDI versus mrCDI secondary bile acids observed at Week 1 were at least an order of magnitude smaller than the percent change in bile acid concentrations from baseline and Week 1 (Figure 2, Supplementary Figure 3).

### Comparison of ECOSPOR IV to ECOSPOR III Placebo-controlled Phase 3 Trial

To contextualize the open-label frCDI and mrCDI patient (ECOSPOR IV) microbiome results, we plotted ECOSPOR IV microbiome data alongside microbiome data from the previously described phase 3 randomized double-blind placebo-controlled trial of VOS composed of only mrCDI patients (ECOSPOR III) to enable visual comparisons [1]. ECOSPOR III baseline and Week 1 active arm samples had consistent alpha and beta diversity as the ECOSPOR IV frCDI and mrCDI subgroups (see Supplementary Figure 5). Similar to the microbiome profiles, bile acid concentrations at baseline and Week 1 in the active arm of ECOSPOR III following VOS dosing were consistent with both frCDI and mrCDI ECOSPOR IV subgroups (see Supplementary Figure 6).

## DISCUSSION

This is one of the largest studies published to date comparing microbiome profiles in patients with frCDI versus mrCDI. The baseline microbiome profiles (collected post-antibiotic and pre-VOS treatment) in both frCDI and mrCDI patients were characterized by hallmarks of a disrupted microbiome that are known to be favorable to *C. difficile* vegetative growth and toxin production: low alpha diversity, higher concentrations of primary bile acids, and lower concentrations of secondary bile acids [8–10].

VOS drug engraftment is the first critical step in driving the pharmacological changes needed to prevent recurrence of *C. difficile*. Significant increases in alpha diversity, including dose species richness, accompanied by large microbiome compositional changes from baseline to 1-week post-VOS treatment, suggest VOS species rapidly engrafted, shifting the microbiome community composition of both frCDI and mrCDI patients. VOS engraftment corresponded with pharmacodynamic changes, specifically declines in primary bile acid concentrations and reciprocal increases in secondary bile acid concentrations in both frCDI and mrCDI patients. We previously showed that VOS treatment following antibacterial therapy for mrCDI resulted in taxonomic and bile acid changes, similar to those described here, and significant differences compared with placebo [1]. These compositional and functional changes are believed to act synergistically to interrupt the two-phase *C. difficile* life cycle.

The changes in microbiome and bile acid profiles from pretreatment baseline to post-VOS dosing at Week 1 were the largest signals in the ECOSPOR IV dataset, regardless of recurrence status. Additionally, most of the head-to-head frCDI versus mrCDI comparisons at baseline and at Week 1 were not statistically significant. The exceptions (baseline dose species richness and Week 1 secondary bile acid concentrations) are unlikely biologically significant given the numerical differences were small, there were large overlaps in distributions of values, and both frCDI and mrCDI patients were accompanied by similarly low CDI recurrence rates at Week 8. These findings suggest that antibiotics followed by a microbiome treatment with VOS may be an optimal treatment strategy for rCDI, regardless of the number of prior episodes.

Guidelines for management of rCDI, which were written prior to the availability of approved live microbiome therapeutics, suggest therapeutic strategies based on the number of recurrences [12, 13]. For those with a first recurrence, an alternate antibiotic is recommended. This strategy is based on the premise that the antibiotic choice was the reason for therapeutic failure; however, both vancomycin and fidaxomicin have excellent bactericidal activity against toxin-producing vegetative *C. difficile* bacteria and achieve high stool concentrations [3]. Neither antibiotic can address the microbiome disruption that characterizes recurrent infections and indeed both can worsen disruption although fidaxomicin has been shown to be generally less disruptive to the microbiome than vancomycin [3, 25]. Comparisons of patients who received vancomycin versus fidaxomicin in this and previous ECOSPOR III analyses suggest that choice of antibiotic has only modest impacts on microbial diversity which are dwarfed by the magnitude of increased diversity after treatment with VOS relative to baseline or placebo [1, 3, 11]. These data suggest that VOS treatment results in both the microbiome composition and microbial metabolic functions necessary to prevent both first recurrence and later recurrence.

While use of investigational FMT in rCDI has been associated with restoration of microbial composition and metabolite production, there is a wide range of reported efficacy in sustained clinical response rates. In a systematic review and meta-analysis, weighted pooled efficacy rates ranged from 68% (95% CI, 54%–81%) in randomized trials to 83% (95% CI, 71%–94%) in open-label trials using FMT [26]. Sustained clinical response rates for VOS were above 90% in both frCDI (93% CI, 85.5%–97.9%) and mrCDI (90.3%, 85.1–94.25) populations in ECOSPOR IV, consistent with the observed efficacy in the placebo-controlled ECOSPOR III trial [5].

Enthusiasm for use of FMT for patients with first recurrence has been tempered due to reports of transmission of undetected pathogens, concerns about emerging pathogens excreted in stool (eg, SARS-CoV-2; Mpox), and theoretical concerns about transmission of disease phenotypes [27]. In contrast, the multi-step manufacturing process for VOS includes solvent (ethanol)-based inactivation of organisms that are not spores and filtration processes with bioburden testing prior to product release that mitigate risks to patients beyond donor screening alone. The final room-temperature stable product of VOS capsules represents ∼1% of donor stool in the purified bacterial spore suspension [28, 29].

There are several limitations of this study. We did not conduct an integrated analysis of the ECOSPOR III and ECOSPOR IV Phase 3 trials due to the possibility of confounding differences in study design and other potential unforeseen biases. Critiques about the limitations of open-label trial design can be applied to the ECOSPOR IV data. Because submission of Week 1 stool samples was optional to not overburden patients, we cannot rule out self-selection bias. However, the observed changes in microbiome composition and microbe-associated functions from baseline to Week 1 in both the frCDI and mrCDI patients in ECOSPOR IV were visually similar to those observed in VOS-treated mrCDI patients in ECOSPOR III [1] (Supplementary Figures 5 and 6), though no formal statistical tests were applied. The strength of this study is the large clinical dataset of frCDI and mrCDI patients, with analysis of multiple elements of the microbiome, which provides evidence that disrupted microbiome composition and function is an underlying pathology in recurrent CDI, regardless of the number of prior recurrences.

## CONCLUSIONS

The microbiome data from this post hoc analysis in patients with first or multiply recurrent CDI suggest commonalities in pathogenesis, specifically antibiotic-induced microbiome compositional and functional disruption, which contribute to future recurrence. These observations suggest the need for microbiome restoration following antibiotics to treat recurrent CDI, regardless of the number of prior CDI episodes, to prevent future recurrences and reduce the morbidity and mortality associated with this debilitating disease.

## Supplementary Material

jiaf408_Supplementary_Data

## Supplementary Data

Supplementary materials are available at *The Journal of Infectious Diseases* online (http://jid.oxfordjournals.org/). Supplementary materials consist of data provided by the author that are published to benefit the reader. The posted materials are not copyedited. The contents of all supplementary data are the sole responsibility of the authors. Questions or messages regarding errors should be addressed to the author.
