# Supplementary material for: Comparability of Gastrointestinal Microbiome and Bile Acid Profiles in Patients With First or Multiply Recurrent Clostridioides difficile Infection
Source: J Infect Dis. 2025 Aug 2;232(5):e733–40. doi: 10.1093/infdis/jiaf408 (PMC12614999; doi:10.1093/infdis/jiaf408)
Supplement: jiaf408_Supplementary_Data [file jiaf408_supplementary_data.zip › frCDI_vs_mrCDI_Supplementary_Figures_Aug_2025.pdf]

Comparability of gastrointestinal microbiome and bile acid profiles in patients with first or multiply recurrent *Clostridioides difficile* infection

SUPPLEMENTAL METHODS

Species profiles were generated from pre-processed, sequence depth normalized WMS data [Diversigen, New Brighton, MN] using MetaPhlAn2 [1] with a proprietary database of species markers for consistency with previous publications [2–4]. PERMANOVA tests were run using the adonis2 function (version 2.6-4) from the vegan package to quantify the variance in community composition explained by time and key subpopulations (Bray-Curtis Distance ~ Timepoint + Antibiotic + Sex + Qualifying CDI episode diagnostic + CDI recurrence status) [5].

Three alpha diversity metrics were calculated with MetaPhlan2 species profiles: the number of unique VOS dose species (dose species richness), the number of unique microbial species (species richness), and the abundance-weighted Shannon diversity metric. Shannon diversity captures differences in both community richness (total number of unique species) and community evenness (the abundance of each species). Drug engraftment was measured as newly appearing dose species at week 1 (i.e., dose species present in week 1 samples that were absent from the same patient's baseline species profile). Bray-Curtis Dissimilarity, a measure of beta diversity, was calculated from MetaPhlan2 species profiles in paired baseline and week 1 patient samples. Bray-Curtis captures the degree to which two samples share the same species at the same abundances.

A quantitative assessment of key primary (cholic and chenodeoxycholic acid) and secondary (deoxycholic, lithocholic, and ursodeoxycholic acid) bile acids in stool were measured with on homogenized, lyophilized stool samples, spiked with internal standards then subjected to liquid-liquid extraction, dilution and liquid chromatography/mass spectrometry (LC-MS/MS) analysis using an Agilent 1290/Sciex 5500 QTRAP system equipped with an Agilent SB-C18 reversed phase column [Metabolon, Durham, NC]. Profiling of bile acids was conducted according to Good Clinical Practice and assay validation included assessment for assay selectivity, specificity, limit of quantification accuracy, and precision and stability. Primary and secondary bile acid pool concentrations were calculated as the summed concentrations of their constituents.

Alpha diversity and bile acid comparisons between frCDI and mrCDI patients were carried out with two-sided Wilcoxon rank-sum tests (MWU), treating antibiotic as a conditional variable using the wilcox_test function in the coin package (R version 3.6.0, [6,7]). Additional PERMANOVA tests were run to interrogate the relationship between Bray-Curtis dissimilarity and frCDI and mrCDI status, controlling for antibiotic by setting strata = antibiotic. Differences in the prevalence and abundance of individual species and genera were interrogated using logistic and linear modeling with antibiotic as a fixed effect, implemented in the MaAslin3 package version 0.99.16 (default settings) [8].

1. Truong DT, Franzosa EA, Tickle TL, et al. MetaPhlAn2 for enhanced metagenomic taxonomic profiling. Nat Methods [Internet]. **2015**; 12(10):902–903.

2. Feuerstadt P, Louie TJ, Lashner B, et al. SER-109, an Oral Microbiome Therapy for Recurrent Clostridioides difficile Infection. New Engl J Med. **2022**; 386(3):220–229.

3. Straub TJ, Lombardo M-J, Bryant JA, et al. Impact of a purified microbiome therapeutic on abundance of antimicrobial resistance genes in patients with recurrent Clostridioides difficile infection. Clin Infect Dis. **2023**; 78(4):833-841.

4. McGovern BH, Ford CB, Henn MR, et al. SER-109, an Investigational Microbiome Drug to Reduce Recurrence after Clostridioides difficile infection: Lessons Learned from a Phase 2 Trial. Clin Infect Dis. **2020**; 72(12):2132–2140.

5. Sims MD, Khanna S, Feuerstadt P, et al. Safety and Tolerability of SER-109 as an Investigational Microbiome Therapeutic in Adults With Recurrent Clostridioides difficile Infection. Jama Netw Open. **2023**; 6(2):e2255758.

6. Hothorn T, Hornik K, Wiel MA van de, Zeileis A. A Lego System for Conditional Inference. Am Stat. **2006**; 60(3):257–263.

7. Kassambara A. rstatix: Pipe-Friendly Framework for Basic Statistical Tests [Internet]. 2023. Available from: https://rpkgs.datanovia.com/rstatix/. Accessed Dec. 2024.

8. Nickols WA, Kuntz T, Shen J, et al. MaAsLin 3: Refining and extending generalized multivariable linear models for meta-omic association discovery. bioRxiv. **2024**; :2024.12.13.628459.

SUPPLEMENTAL FIGURES

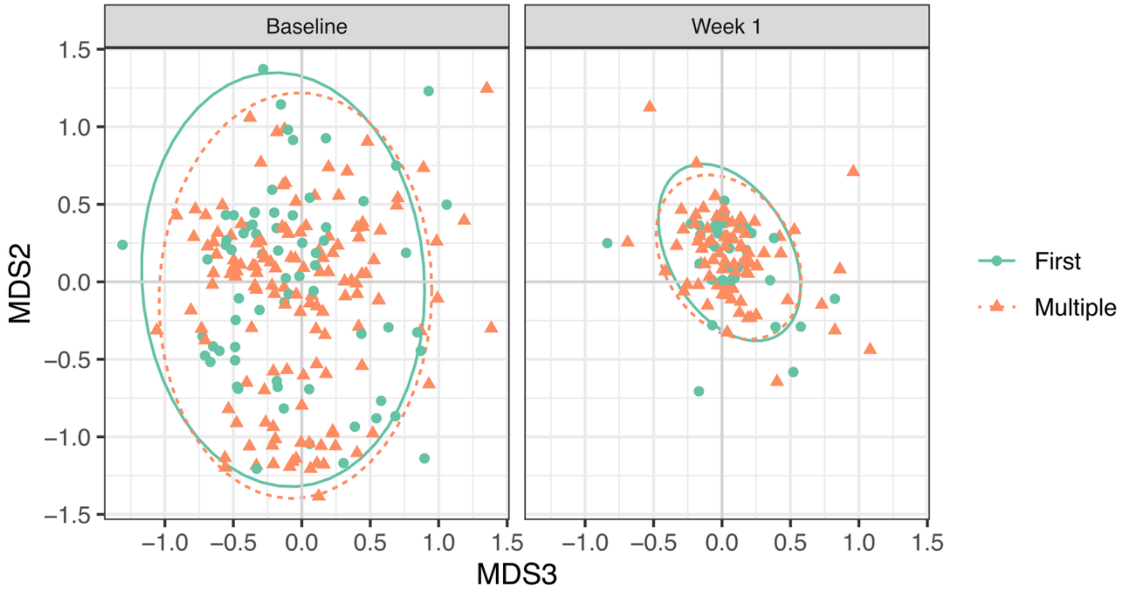

Supplementary Figure 1. Axes 2 and 3 of nonmetric multidimensional scaling (NMDS) plot displayed in Figure 1A.

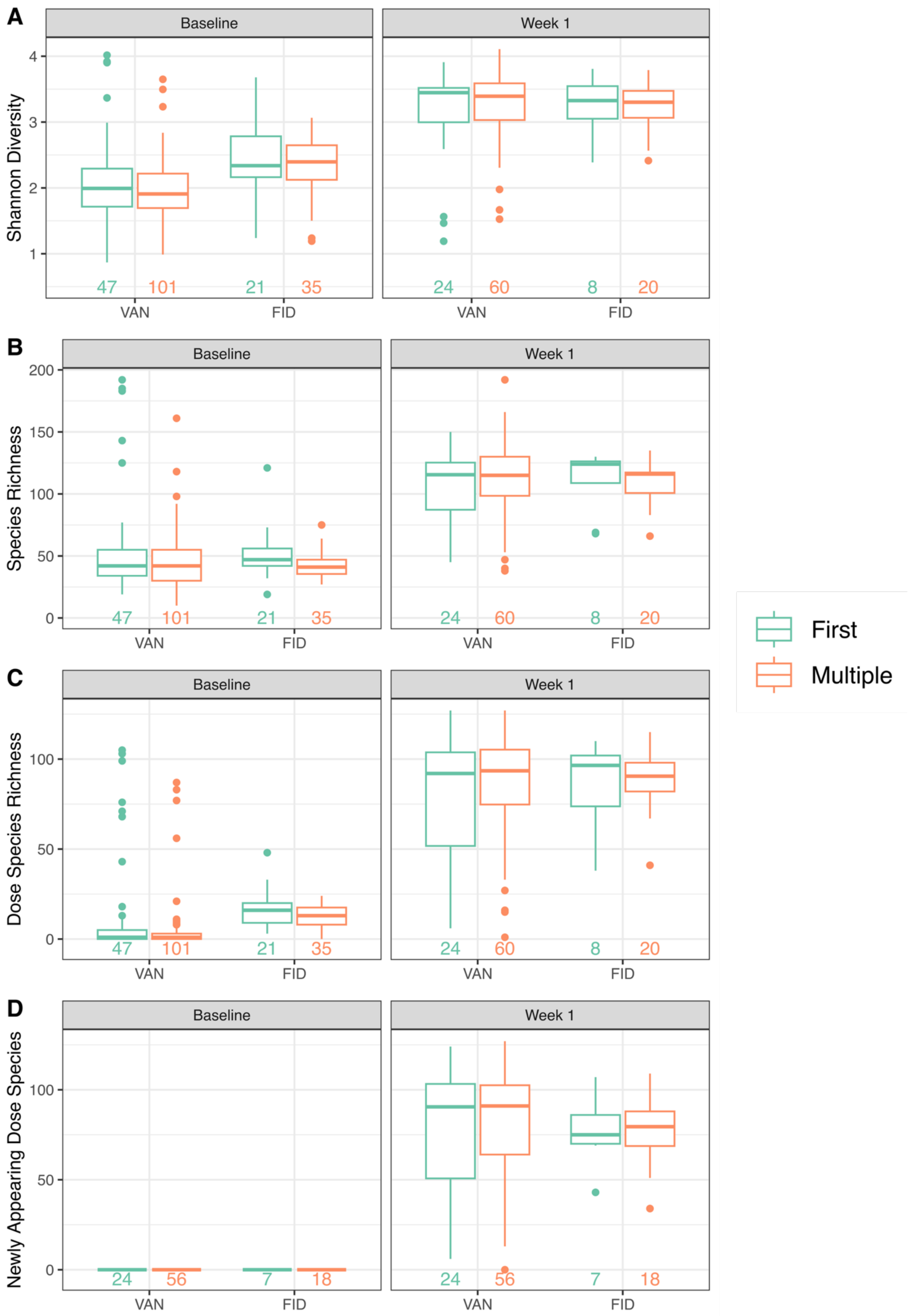

A
Baseline
Week 1
Shannon Diversity
47
101
21
35
24
60
8
20
VAN
FID
B
Species Richness
C
Dose Species Richness
D
Newly Appearing Dose Species
24
56
7
18
First
Multiple

Supplementary Figure 2. Comparison of Shannon diversity (A) species richness (B) dose species richness (C) and newly appearing dose species (D) from first (frCDI green) and multiply (mrCDI, orange) rCDI patients treated with VOS at Baseline and Week 1. Samples are grouped on the x-axis by standard-of-care (SoC) antibiotics completed for the treatment of the CDI just prior to VOS dosing. Sample numbers are displayed on the bottom of each plot.

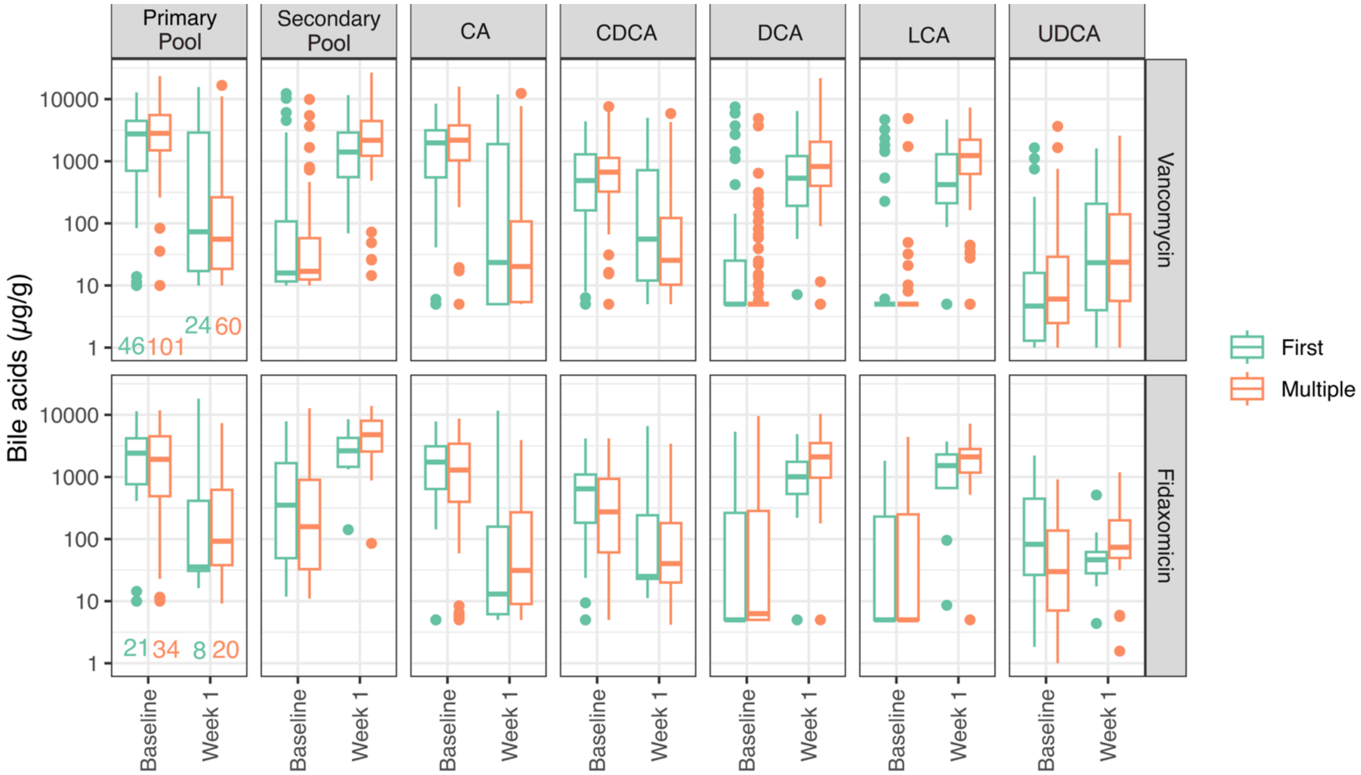

Supplementary Figure 3. Comparison of pooled and individual primary (cholic acid (CA) and chenodeoxycholic acid (DCA)) and secondary (lithocholic qcid (LCA), deoxycholic acid (DCA), and ursodeoxycholic acid (UDCA)) bile acids concentrations (µg/g dried stool) from first (frCDI, green) and multiply (mrCDI, orange) rCDI patients at baseline and week 1. Prior to providing a baseline sample, patients were treated with either standard-of-care vancomycin (VAN) or fidaxomicin (FID) for their qualifying episode of rCDI, with patients receiving each antibiotic plotted separately. Sample numbers are displayed on the bottom of the primary pooled bile acid plot.

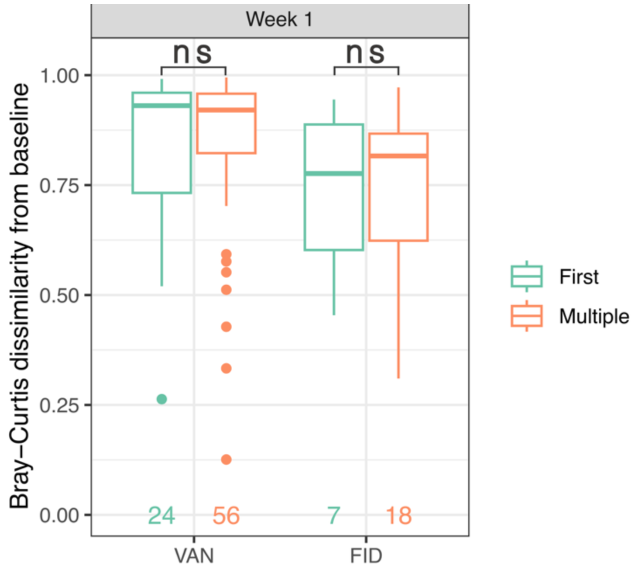

Supplementary Figure 4. Comparison of change in community composition (Bray-Curtis dissimilarity) from patient's baseline to week 1 sample, within first (frCDI, green) and multiply (mrCDI, orange) patients. Prior to providing a baseline sample, patients were treated with either standard-of-care vancomycin (VAN) or fidaxomicin (FID) for their qualifying episode of rCDI, with patients receiving each antibiotic plotted separately (x-axis). Sample numbers are displayed on the bottom of the plot.

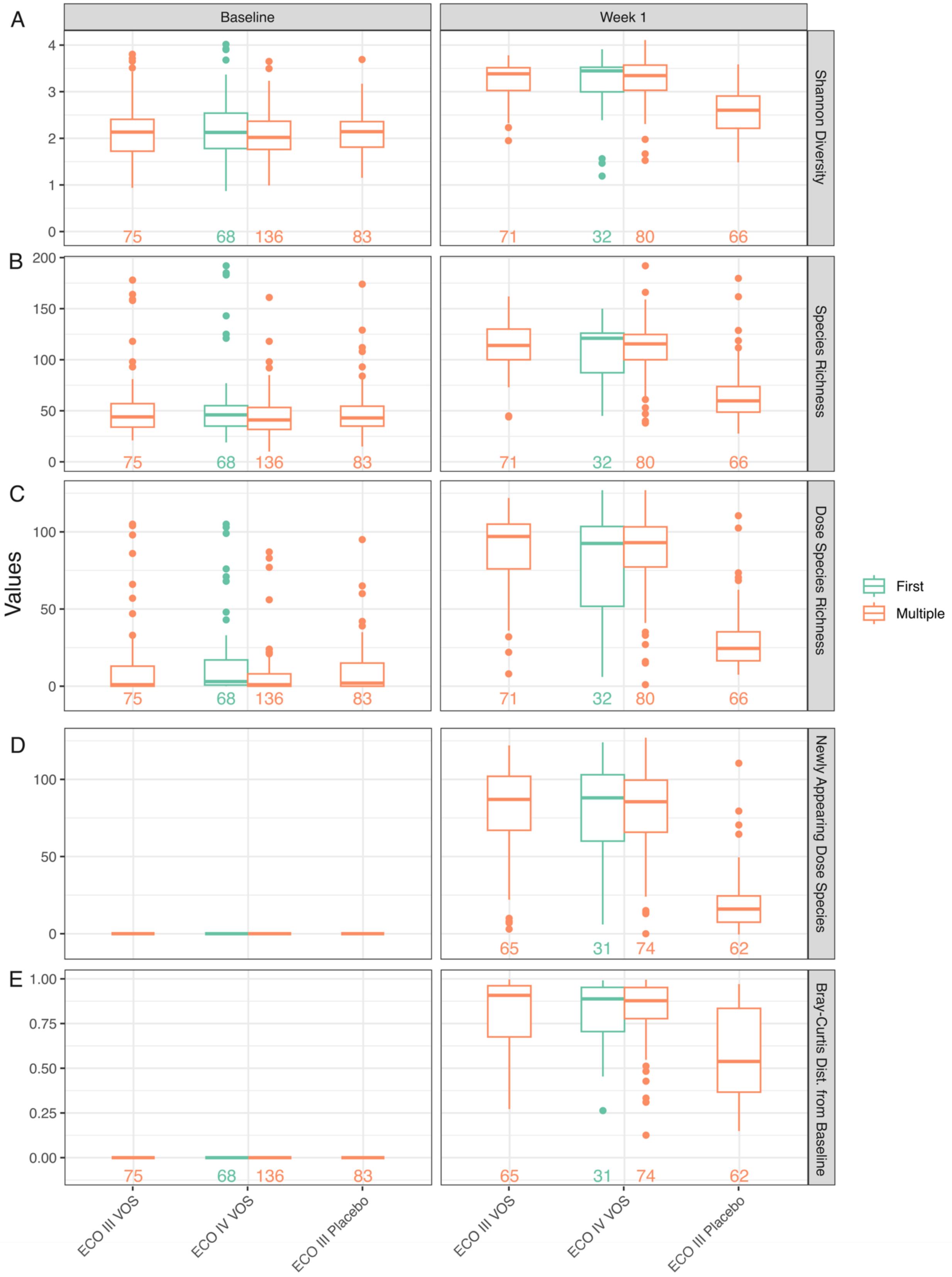

A
B
C
D
E
Baseline
Week 1
Shannon Diversity
Species Richness
Dose Species Richness
Newly Appearing Dose Species
Bray-Curtis Dist. from Baseline
Values
First
Multiple
ECO III VOS
ECO IV VOS
ECO III Placebo
75
68
136
83
71
32
80
66
65
31
74
62

Supplementary Figure 5. Juxtaposition of microbiome diversity metrics from patients in the double-blind randomized control ECOSPOR III study (ECO III), which only enrolled multiply rCDI patients and the single-arm open-label ECOSPOR IV study (ECO IV), which enrolled both first (frCDI) and multiply(mrCDI) patients. A) Shannon diversity, B) species richness, C) dose species richness, D) newly appearing dose species and E) Bray-Curtis distances are plotted for both VOS arms (ECOSPOR III & IV) and the placebo arm (ECOSPOR III). Newly appearing dose species and E) Bray-Curtis distances are both relative to baseline timepoints. Numbers on bottom of figure indicate the number of samples in each box. ECOSPOR III data shown in this figure is adapted from Feuerstadt et al. New Engl J Med. 2022; 386(3):220–229[2]. All available ECOSPOR III samples that passed quality criteria were included in this analysis.

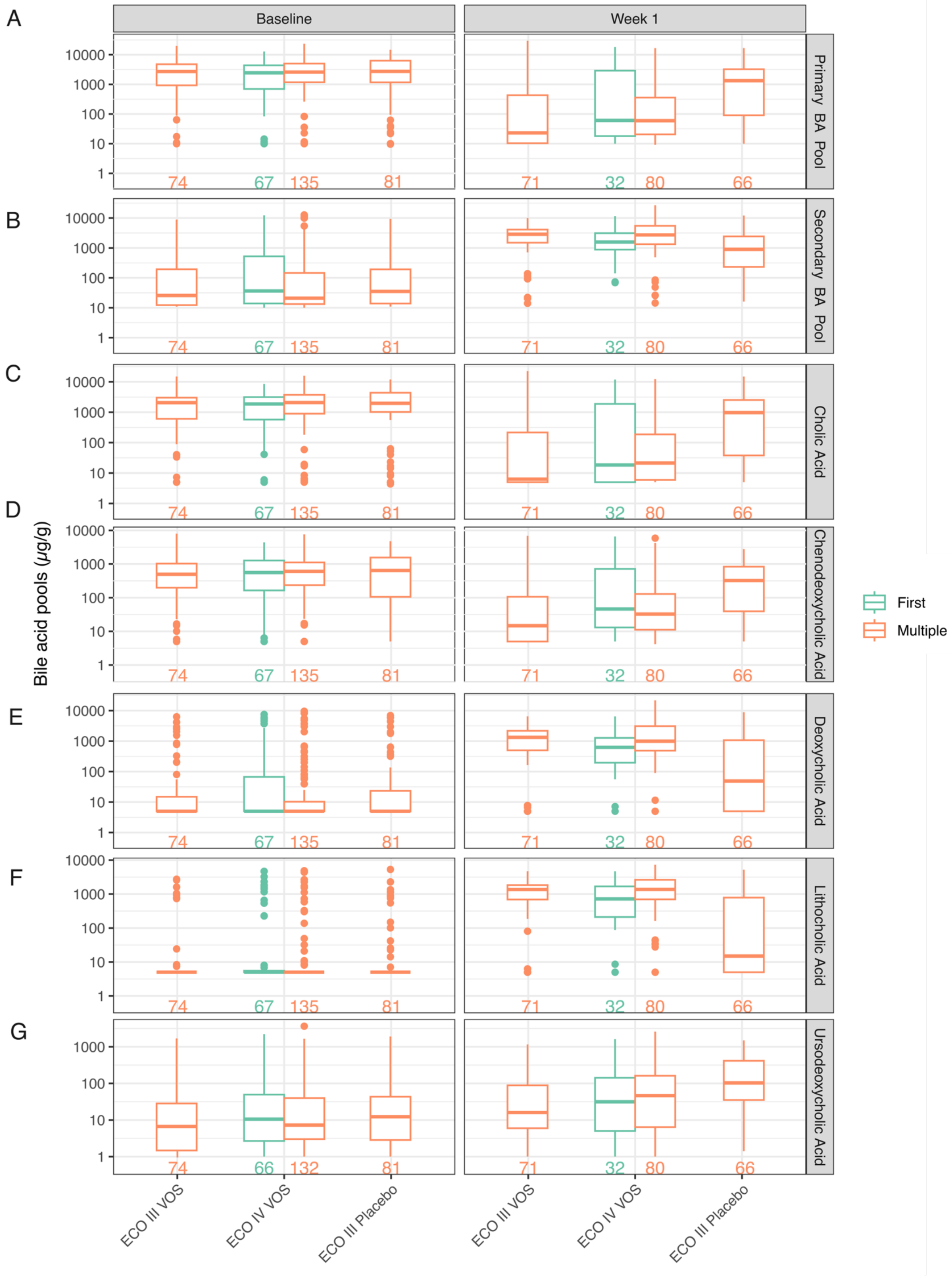

A
B
C
D
E
F
G
Baseline
Week 1
Primary BA Pool
Secondary BA Pool
Cholic Acid
Chenodeoxycholic Acid
Deoxycholic Acid
Lithocholic Acid
Ursodeoxycholic Acid
Bile acid pools (μg/g)
10000
1000
100
10
1
74
67
135
81
71
32
80
66
66
132
First
Multiple
ECO III VOS
ECO IV VOS
ECO III Placebo

Supplementary Figure 6. Juxtaposition of bile acid concentration from patients in the double-blind randomized control ECOSPOR III study (ECO III), which only enrolled multiply rCDI patients (mrCDI) and the single-arm open-label ECOSPOR IV study (ECO IV), which enrolled both first (frCDI) and multiply (mrCDI) rCDI patients. **A)** Pooled primary bile acids, B) pooled secondary bile acids and individual bile acids C) cholic acid, D) chenodeoxycholic acid, E) deoxycholic acid F) lithodeoxycholic acid and G) ursodeoxycholic acid are plotted for both VOS arms (ECOSPOR III & IV) and the placebo arm (ECOSPOR III). Numbers on bottom of figure indicate the number of samples in each box. ECOSPOR III data shown in this figure is adapted from Feuerstadt et al. New Engl J Med. 2022; 386(3):220–229[2]. All available ECOSPOR III samples that passed quality criteria were included in this analysis.
